# Orthotopic Heart Transplantations in a Newly Opened Transplantation Center in Poland: Population Description and Prognosis

**DOI:** 10.3390/jcm15218229

**Published:** 2026-10-22

**Authors:** Mateusz Sokolski, Jakub Ptak, Michał Wilk, Wiktoria Zychla, Paulina Tomecka, Maciej Szwajkowski, Małgorzata Makieła, Magdalena Cielecka, Mateusz Rakowski, Maciej Bochenek, Roman Przybylski, Michał Zakliczyński

**Affiliations:** 1 Institute of Heart Diseases, University Hospital in Wrocław, 50-556 Wroclaw, Poland; 2 Clinic of Cardiac Transplantation and Mechanical Circulatory Support, Institute of Heart Diseases, Wroclaw Medical University, 50-556 Wroclaw, Poland; 3 Institute of Heart Diseases, Student Scientific Club of Transplantology and Advanced Therapies of Heart Failure, Wroclaw Medical University, 50-556 Wrocław, Poland

**Keywords:** heart transplantation, advanced heart failure, heart transplant outcomes, mortality, prognostic factors, central venous pressure, donor–recipient sex mismatch

## Abstract

**Introduction:** Heart transplantation (HTx) remains the definitive treatment for selected patients with advanced heart failure. The early post-transplant period is characterized by substantial morbidity and requires intensive surveillance. The aim of this study was to describe the clinical course of the first 100 heart transplant recipients treated at our newly established center, with particular emphasis on in-hospital outcomes and the 12-month follow-up. **Methods**: We retrospectively analyzed 100 consecutive patients who underwent orthotopic HTx between February 2021 and May 2023 at our center. Demographic, clinical, hemodynamic, laboratory, treatment, and post-transplant follow-up data were collected. The follow-up assessment included survival, rejection, infectious complications, renal replacement therapy (RRT), heart failure decompensation, hospitalizations, outpatient visits, and changes in pharmacotherapy. Cox proportional hazards regression and Receiver Operating Characteristic (ROC) analyses were also performed to assess factors associated with 12-month mortality. **Results:** The study population comprised 82 (82%) men and 18 (18%) women with a median age of 54 years (IQR, 45–64). The in-hospital course was characterized by substantial early morbidity: 17 patients (17%) died during the index hospitalization, with a median time to death of 17 days (IQR, 3–78). RRT was required in 44% of patients. Among hospital survivors, the median length of hospital stay was 43 days and the median ICU stay was 7 days. During the 12-month follow-up, clinically significant cellular graft rejection (grade ≥ 3) occurred at least once in 51% of patients, infectious complications in 45%, and one patient underwent retransplantation. The twelve-month all-cause mortality was 23%. Patients had a mean of 8 planned hospitalizations, 0.4 unplanned admissions, and 1.3 outpatient visits per patient. Exploratory analyses identified possible associations between selected pre-transplant variables and 12-month mortality. **Conclusions:** The first 100 HTx recipients treated at the newly established center experienced a clinically demanding in-hospital course, with a 17% in-hospital mortality. After discharge, the first year remained characterized by frequent cellular rejection incidence, and infectious complications. The twelve-month survival was 77%. These findings provide a comprehensive description of the early in-hospital and 12-month clinical course of a newly established Htx program.

## 1. Background

Advanced heart failure (AdHF) poses a significant public health challenge, particularly in the context of the increasing incidence and prevalence of heart failure (HF) in the aging Polish population. Despite substantial advances in therapeutic options, HF may progress to advanced stages, highlighting the need for effective advanced treatment strategies [1,2,3].

Heart transplantation (HTx) remains the gold standard for managing AdHF, offering the potential for improved survival and quality of life [4]. The identification of suitable candidates for HTx involves a combination of clinical assessment, cardiopulmonary exercise testing (CPET), right heart catheterization (RHC), and prognostic evaluation tools [5].

In Poland, heart transplantation efforts are coordinated by Poltransplant, the national transplant organization. In 2021, Poland achieved a milestone with 200 HTx ranking eighth among European Union nations in terms of transplant volume [6]. However, the number of transplants subsequently declined to 171 in 2022, followed by 175 in 2023, 201 in 2024, and 169 in 2025. As of 2025, a mean of 375 patients remains on the active waiting list, highlighting a growing gap between organ availability and clinical needs. This disparity highlights the importance of strengthening donor programs and increasing public awareness of enhancing organ donation.

Eligibility for HTx and organ allocation hinges on accurately predicting post-transplant survival, often through multivariable regression models [7]. A comprehensive evaluation of transplant candidates involves assessing the severity and trajectory of extra-cardiac conditions, with particular focus on malnutrition and frailty as critical prognostic factors [5].

Post-transplant survival is influenced by a complex interplay of modifiable and non-modifiable factors [8]. During the first year following HTx, infections, HF due to the graft rejection, and multi-organ failure are the leading causes of mortality, whereas longer-term risks include malignancies, vascular graft disease, and recurrent HF.

Given the increased risk of mortality during the first year after transplantation, a detailed analysis of recipient-related factors influencing outcomes was conducted. This study aimed to describe our center’s experience with the first 100 heart transplants, including the baseline characteristics, in-hospital course, and clinical outcomes during 12 months of follow-up. As a secondary, exploratory objective, we assessed recipient-related factors associated with in-hospital and 12-month mortality.

## 2. Material and Methods

### 2.1. Study Population

In our study, we retrospectively analyzed the first 100 consecutive patients who underwent HTx at our center between 25 February 2021, and 18 May 2023. All patients were followed for 12 months or until death. Patients who survived beyond 12 months were censored at 365 days. Patient data were obtained from the local HTx registry, which systematically records comprehensive medical information during the hospitalization period and subsequent scheduled follow-up visits. Patients were followed for 12 months after transplantation or until death, whichever occurred first. Survivors were censored at 365 days. The qualification process included an assessment of disease severity, functional capacity, hemodynamic status, and overall prognosis, with relevant parameters including cardiopulmonary exercise testing and pulmonary hemodynamics. Patients who did not meet the established criteria for heart transplantation were not qualified for transplantation and were therefore not included in the study cohort. The study population comprised the first 100 consecutive patients who met the transplantation criteria and subsequently underwent transplantation during the study period. No patient who met the inclusion criteria and underwent HTx was subsequently excluded from the analysis.

### 2.2. Clinical, Laboratory, and Hemodynamic Parameters

Demographic, clinical, hemodynamic, laboratory, treatment, perioperative, and follow-up data were collected from the institutional heart transplantation registry and medical records. Baseline assessment included demographic characteristics, underlying heart failure etiology, comorbidities, previous cardiovascular interventions, pre-transplant mechanical circulatory support, hemodynamic parameters obtained during right heart catheterization, and laboratory parameters measured before transplantation. Perioperative data included selected treatment and clinical parameters during the index hospitalization. Post-transplant follow-up data included mortality, rejection episodes, infectious complications, heart failure decompensation, renal replacement therapy, retransplantation, hospitalizations, outpatient visits, and selected clinical parameters.

### 2.3. Endomyocardial Biopsy Protocol and Rejection Assessment

Surveillance endomyocardial biopsies were performed according to the institutional post-transplantation protocol. During the first month after transplantation, four biopsies were performed at weekly intervals. During the second month, two biopsies were performed at two-week intervals. One biopsy was subsequently performed during the third month and one during the fourth month, followed by biopsies every two months thereafter, resulting in a total of 12 surveillance biopsies during the first year after transplantation.

Acute cellular rejection was assessed according to the International Society for Heart and Lung Transplantation (ISHLT) grading system. Clinically significant cellular rejection was defined as grade ≥ 3. For the 12-month analysis, patients were classified as having experienced rejection if at least one grade ≥ 3 episode was documented during follow-up; multiple episodes in the same patient were counted as a single patient-level outcome.

### 2.4. Follow-Up and Outcome Assessment

During the 12-month post-transplant follow-up, clinical status was assessed at predefined follow-up time points. Data on outpatient visits, planned and unplanned hospitalizations, body weight, BMI, heart rate, and blood pressure were collected. Major post-transplant complications, including acute cellular rejection, infectious complications, heart failure decompensation, renal replacement therapy, and retransplantation, were recorded throughout the follow-up period.

### 2.5. Endpoints

The primary endpoints were in-hospital all-cause mortality and 12-month all-cause mortality. Secondary outcomes included clinically significant acute cellular rejection (grade ≥ 3), infectious complications, heart failure decompensation, renal replacement therapy, retransplantation, and healthcare utilization during the 12-month follow-up period.

### 2.6. Statistical Analysis

Normally distributed continuous variables were presented as mean ± standard deviation (SD), whereas non-normally distributed variables were presented as median and interquartile range (IQR). The normality of the variable distribution was assessed using the Shapiro–Wilk test. To explore intergroup differences, the Student’s t-test was applied for normally distributed variables, while the Mann–Whitney U test was employed for variables with a skewed distribution. Categorical variables were succinctly presented as numbers accompanied by percentages. Differences in categorical variables between the compared groups were assessed using the chi-square test. A significance threshold of *p* < 0.05 was applied. The primary endpoints of this study were in-hospital all-cause mortality and 12-month all-cause mortality.

Cox proportional hazards regression models were used to estimate hazard ratios (HRs) and corresponding 95% confidence intervals (CIs). Variables that differed significantly between 12-month survivors and non-survivors were subsequently evaluated in Cox proportional hazards regression models, specifically: HF ischemic etiology, donor-recipient sex mismatch, pre-procedural central venous pressure (CVP), pre-procedural troponin level, and pre-procedural albumin level. A donor–recipient sex mismatch was defined as positive when a male recipient received a female donor heart. Given the exploratory nature of the study and the relatively small number of deaths, the number of variables included in the Cox proportional hazards analyses was deliberately limited. Included variables were initially identified based on statistically significant differences between patients who survived for 12 months and those who died during that period. This approach was adopted as a pragmatic strategy to limit the number of covariates included in multivariable analyses and to avoid model overfitting within this relatively small cohort. The Cox regression analyses performed were considered exploratory and hypothesis-generating rather than confirmatory.

Receiver operating characteristic (ROC) curve analysis was performed to determine optimal cut-off values for pre-procedural CVP, troponin, and albumin.

All statistical analyses were conducted using the STATISTICA 13 data analysis software system (StatSoft Polska Sp. z o.o. 2024. Zestaw Plus version 5.0.96. www.statsoft.pl).

## 3. Results

### 3.1. Characteristics of the Study Population

The study population comprised 82 (82%) men and 18 (18%) women, with a median age of 54 years (IQR 45–64). Ischemic HF etiology was present in 44% of patients, while arterial hypertension and hyperlipidemia were present in 46% and 41%, respectively. Blood type A was the most common (45%), followed by blood type O (23%). Hemodynamic measures showed a median of CVP 9.0 mmHg, pulmonary artery mean pressure 30.9 mmHg, and median cardiac output of 4.1 L/min. The median pulmonary vascular resistance was 2.3 Wood units; 17 patients required reversibility testing due to the high pulmonary vascular resistance (PVR). Laboratory testing performed one week before HTx showed generally preserved blood counts and renal function, with elevated N-terminal pro-B-type natriuretic peptide (NT-proBNP) levels. Baseline laboratory parameters are presented in Table 1.

### 3.2. Treatment

Before HTx, 20% of patients required temporary MCS, while 7% had a durable LVAD (HeartMate 3) in place. Most patients (82%) were receiving beta-blockers, 70% angiotensin-converting-enzyme inhibitor/angiotensin II receptor blocker or sacubitril/valsartan, and 70% were prescribed mineralocorticoid receptor antagonists (MRAs). Anticoagulation therapy was administered to 68%, and 58% were on statins.

### 3.3. In-Hospital Course

Seventeen patients (17%) died during the index hospitalization. The median time to death was 17 days (IQR 3–78 days). Causes of death included left-sided or biventricular HF (41%), isolated right ventricular failure (12%), hemorrhage (12%), sepsis (23%), and other causes (12%). RRT was required in 44% of patients. For survivors, the median hospital stay was 43 days, with a median intensive care unit (ICU) stay of 7 days (Table 2). One week after HTx, laboratory results showed an elevated white blood cell count (WBC) at a median of 17.5 × 10^3^/μL, as well as significantly higher levels of troponin (8175 ng/L) and C-reactive protein (CRP, 48.5 mg/L). By one month post-transplant, WBC had decreased to a median of 10.7 × 10^3^/μL, and there were improvements in renal function, with a reduction in creatinine levels (1.08 mg/dL) and an increase in albumin (3.20 g/dL) (Table 3).

### 3.4. Out-of-Hospital Course

Clinical outcomes during the 3-, 6-, and 12-month follow-up periods are summarized in Table 4. In the 12-month follow-up, the average number of planned hospitalizations per patient was 8, with 0.4 unplanned admissions and 1.1 outpatient visits per patient.

Rejection episodes were observed throughout the post-transplant follow-up period. Clinically significant acute cellular rejection (grade ≥ 3) was documented in 15 patients during the first week after transplantation and in 26 patients at the 1-month assessment. Subsequently, grade ≥ 3 acute cellular rejection was documented in 16 patients at 3 months, 8 patients at 6 months, and 7 patients at 12 months. For this patient-level outcome, repeated rejection episodes in the same patient were counted only once. Accordingly, 51 patients (51%) experienced at least one episode of clinically significant acute cellular rejection (grade ≥ 3 according to the ISHLT classification) during the 12-month follow-up period.

**Figure 1:**
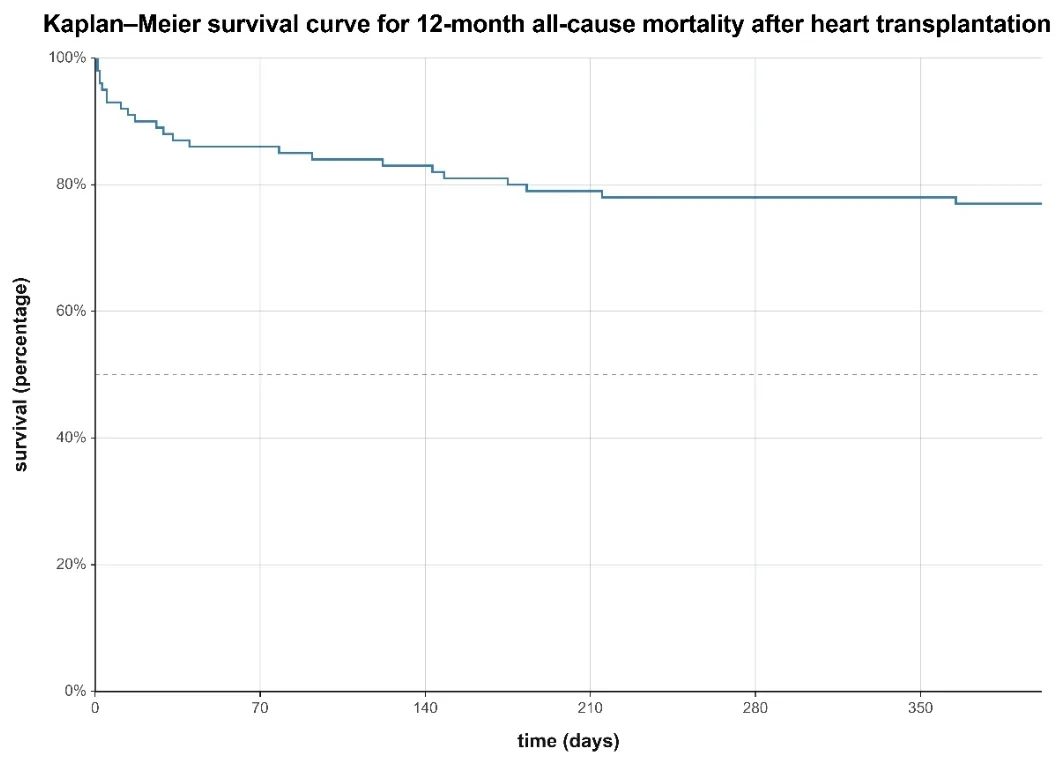
Kaplan–Meier survival curve for 12-month all-cause mortality after heart transplantation.

Over the 12-month follow-up period, infectious complications occurred in 45% of patients, 6% required renal replacement therapy, and 5% experienced heart failure decompensation. Mortality at 12 months was 23%, with a median time to death of 31 days (5–132.5 days), and 1 patient underwent re-transplantation (re-HTX) after 103 days from primary Htx. The 12-month survival is demonstrated in Figure 1.

During the post-transplant follow-up, patients received standard immunosuppressive therapy based predominantly on tacrolimus, mycophenolate mofetil, and corticosteroids, with minor changes in the immunosuppressive regimen over time. Cardiovascular and supportive pharmacotherapy was also adjusted according to the patients’ clinical status. The use of immunosuppressive and selected concomitant medications at 3, 6, and 12 months after HTx is summarized in Table 5. These parameters were evaluated during subsequent follow-up periods and are summarized in Table 6.

### 3.5. Exploratory Analysis of Factors Associated with 12-Month Mortality

**Figure 2:**
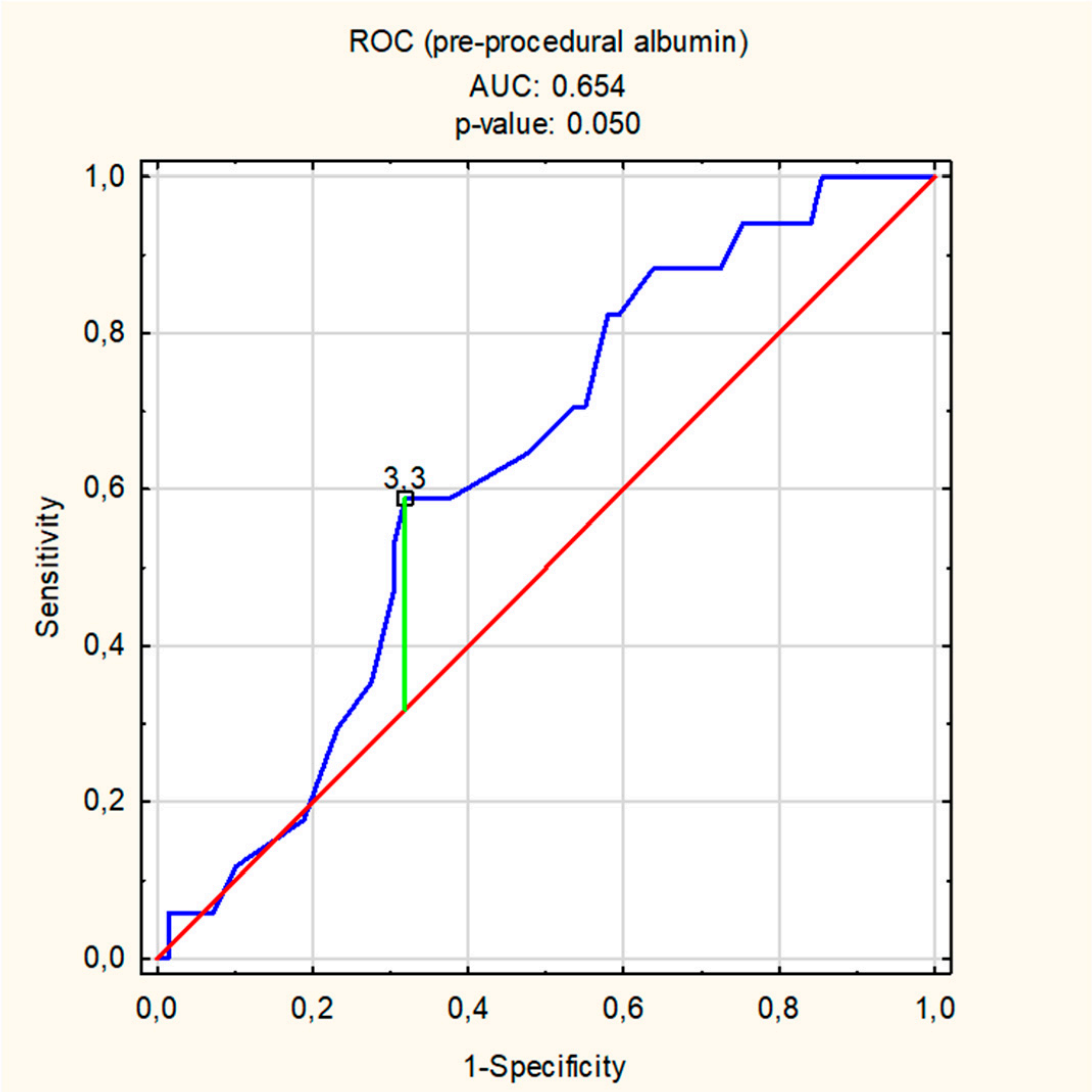
Receiver operating characteristic (ROC) curve for pre-transplant serum albumin in predicting 12-month all-cause mortality after heart transplantation. The area under the curve (AUC) was 0.654 (*p* = 0.050). The identified cut-off value for pre-transplant albumin was 3.3 g/dL. In the figure, the blue line represents the ROC curve, the red diagonal line serves as a reference line corresponding to random classification, and the green line indicates the selected cutoff point, along with its corresponding sensitivity and false-positive rate (1 − specificity) values.

**Figure 3:**
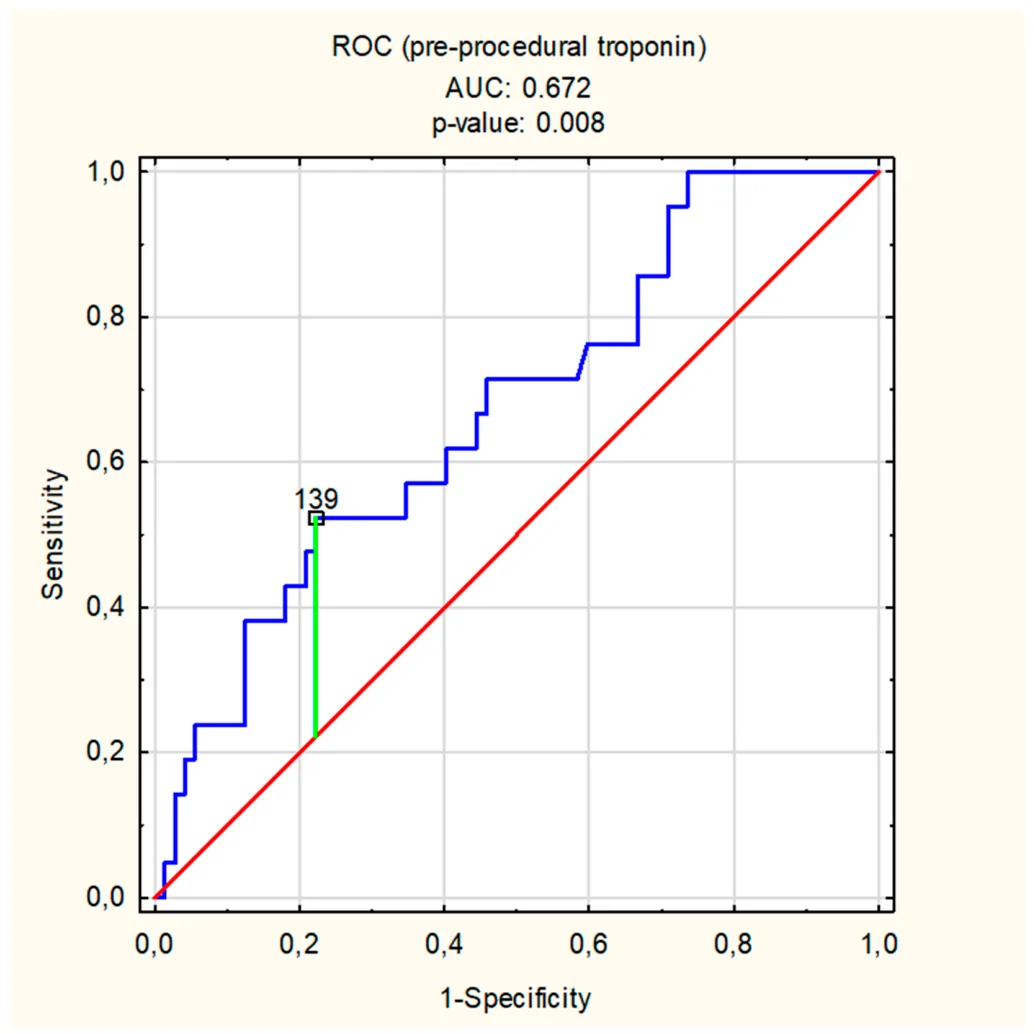
Receiver operating characteristic (ROC) curve for pre-transplant troponin level in predicting 12-month all-cause mortality after heart transplantation. The area under the curve (AUC) was 0.672 (*p* = 0.008). The identified cut-off value for pre-transplant troponin was 139 ng/L. In the figure, the blue line represents the ROC curve, the red diagonal line serves as a reference line corresponding to random classification, and the green line indicates the selected cutoff point, along with its corresponding sensitivity and false-positive rate (1 − specificity) values.

Several variables differed significantly between 12-month survivors and non-survivors, namely, ischemic HF etiology, female-donor-to-male-recipient sex mismatch, pre-procedural CVP, pre-procedural troponin level, and pre-procedural-albumin level. Therefore, we created Cox regression models to explore potential associations between these variables and mortality within one year post-HTx. The models are presented in Table 7. The ROC curves analysis showed the cut-off values for the above parameters (Figure 2 and Figure 3).

## 4. Discussion

This study describes the initial experience of a newly established heart transplantation center, focusing on the clinical trajectory of the first 100 recipients from the perioperative period through 12 months of follow-up. In-hospital mortality was 17%, while 12-month mortality was 23%, indicating that most deaths occurred early after transplantation. The first year was also characterized by substantial morbidity and healthcare utilization, including cellular rejection of at least grade 3 in 51% of patients, infectious complications in 45%, and frequent planned hospitalizations. The relatively high frequency of clinically significant cellular rejection may partly reflect the intensive surveillance biopsy protocol used during the first year after transplantation, particularly during the early post-transplant period, which increases the likelihood of detecting clinically significant rejection episodes. Similarly, the high frequency of infectious complications may reflect the intensive immunosuppressive regimen and the high susceptibility to infection during the early post-transplant period.

The data obtained during the analysis were used to identify potential factors associated with mortality and to compare them with data available from other registries. Accordingly, higher pre-procedural CVP, troponin levels and female-donor-to-male-recipient sex mismatch were associated with a higher 12-month mortality; however, these findings should be considered hypothesis-generating rather than evidence of established prognostic factors, given the small number of deaths, the single-center design, and the exploratory nature of the analyses, which require validation in larger multicenter cohorts.

The International Society for Heart and Lung Transplantation (ISHLT) registry provides a global benchmark for comparing heart transplant practices. In our center, the median recipient age was 54 years (IQR 45–64), similar to the ISHLT cohort’s mean of 55 years (48–68), while the proportion of male recipients was higher (82% vs. 74%).

Notably, our cohort has a higher prevalence of chronic kidney disease (CKD) (30% vs. 4.6%), a known risk factor for poor transplant outcomes. In the literature, the presence of CKD before HTx was identified as a risk factor associated with an increased risk of developing malignant tumors after HTx [9].

Our cohort also exhibited an elevated pulmonary pressure burden, with a mean pulmonary artery pressure (mPAP) of 30.9 mmHg.

Additionally, fewer patients in our cohort received MCS (27% vs. 40.4%), which may reflect differences in treatment strategies, patient selection, and local practice [10,11]. Further research is needed to assess the impact of MCS use on post-transplant outcomes across centers with different protocols [12].

In a study analyzing UNOS data from 2018 to 2022 (n = 11,489), older age, female sex, blood group O, heart failure etiology, body mass index (BMI), mean pulmonary arterial pressure, creatinine, bilirubin, and listing with left-ventricular assist device (LVAD), extracorporeal membrane oxygenation (ECMO), or a ventilator were all associated with a higher post-transplant mortality. In the test cohort, 1-year survival rates were 95%, 91%, 82%, and 66% for low-, moderate-, high-, and very high-risk groups, respectively [13].

In our cohort, elevated troponin levels were associated with increased mortality, aligning with findings by Park et al. (2017) [14], who demonstrated that even mild preoperative troponin elevations were linked to higher 30-day mortality following non-cardiac surgeries, with a hazard ratio (HR) of 1.73 (95% CI: 1.39–2.16, *p* < 0.001) [15]. Similar findings have been reported in other studies showing that elevated preoperative cardiac troponin is associated with increased postoperative morbidity and cardiovascular mortality [15,16]. Moreover, preoperative hs-cTnI has been strongly linked to one-year postoperative mortality following liver transplantation [14]. M’Pembele and Roth (2023) also found that postoperative high-sensitivity troponin T (hsTnT) levels at 48 h post-heart transplantation were strong predictors of one-year mortality and days alive and out of hospital (DAOH), with higher levels correlating to increased mortality (OR = 8.10, 95% CI: 2.14–30.87) [17]. These findings reinforce the importance of monitoring troponin levels both pre- and post-transplant as a key biomarker for predicting complications and mortality. However, the prognostic significance of troponin may vary depending on the clinical context, timing of measurement, underlying heart failure etiology, and the presence of concomitant factors that may increase troponin concentrations, such as renal dysfunction or hemodynamic instability. Therefore, the association observed in our cohort should be interpreted cautiously and considered hypothesis-generating.

Our findings regarding hypoalbuminemia (<3.0 g/dL) are consistent with those of M’Pembele and Roth (2024), who demonstrated that low albumin levels were independently associated with increased 1-year mortality and reduced DAOH (OR = 4.76, 95% CI: 1.94–11.67) [18]. This supports the role of hypoalbuminemia as an important prognostic marker for poor outcomes in heart transplant patients.

Further analysis of a cohort of 822 heart transplant patients identified several other predictors of poor survival, including albumin < 3.5 g/dL, renal dysfunction, and prior cerebrovascular accidents [19]. These findings reinforce the need for comprehensive risk stratification in heart transplant patients. In 743 HTx patients at a single center (1999–2010), those who died within 3 years (n = 153) had lower serum albumin (3.7 ± 0.7 vs. 4.0 ± 0.6 mg/dL, *p* < 0.0001) and higher bilirubin levels (1.4 ± 1.8 vs. 1.1 ± 0.8 mg/dL, *p* = 0.0087) compared to survivors (n = 513) [20].

The International Heart Transplant Survival Algorithm (IHTSA) has demonstrated that a female donor–male recipient mismatch is associated with poorer survival outcomes, with donor sex being a significant factor influencing transplant success [21]. Similar trends have been observed in other organ transplants, such as kidney and liver, where gender-based differences in immunogenicity may contribute to these disparities [22]. According to a meta-analysis, sex mismatch—specifically female donor to male recipient—was significantly linked to increased mortality within the first year post-transplant, with a hazard ratio of 1.38 (95% CI 1.06 to 1.80, high quality) [23].

In our analysis, mismatch as a covariate in proportional hazards regression models consistently showed an increased risk of poor survival. In Model 3, mismatch had *p* = 0.026, with a hazard ratio of 2.83 (95% CI: [1.13, 7.11]), and, in Model 4, mismatch had *p* = 0.029 with a hazard ratio of 2.74 (95% CI: [1.11, 6.77]). These findings highlight the significant impact of donor–recipient sex mismatch on heart transplant survival.

In 517 consecutive adult patients who underwent HTx, CVP was identified as an independent predictor of in-hospital mortality, with an odds ratio of 1.09 (95% CI 1.03–1.15, *p* = 0.004), emphasizing its significant role in influencing patient outcomes [24]. According to the literature, the optimal hemodynamic values for the donor include a CVP of 8–12 mmHg [25]. In our study, pre-procedural CVP demonstrated a moderate predictive value for mortality, with an area under curve (AUC) of 0.707 (*p* = 0.011). Therefore, it is essential in patients’ optimization prior to Htx to stabilize the hemodynamic status through inotropic therapy and appropriate volume regulation using tailored doses of diuretics, provided their clinical condition allows for it [26].

## 5. Limitations

This study has several limitations. First, the retrospective, single-center design and relatively small sample size limit the generalizability of the results.

Second, only 23 deaths were recorded during the 12-month follow-up, which limits the statistical power of survival analyses and may lead to unstable estimates when multiple prognostic variables are considered. Therefore, the identified associations should be considered exploratory and interpreted with caution.

Third, the study includes the first 100 heart transplants performed at our institution; therefore, the observed results may partially reflect the learning curve associated with establishing a new transplant program, as well as changes in patient selection and perioperative management over time.

Furthermore, the selection of variables for Cox regression was based on differences observed between survivors and non-survivors and therefore may have introduced data-driven selection bias. Given the limited number of mortality events, the multivariable analyses should be interpreted as exploratory and hypothesis-generating. Larger multicenter cohorts with a greater number of outcome events are needed to establish robust and clinically validated prognostic models after heart transplantation.

Finally, the cut-off values identified in the ROC analysis for preprocedural CVP, troponin, and albumin were derived from this relatively small cohort and were not externally validated. These cut-offs should therefore be considered hypothesis-generating rather than definitive clinical cut-offs and require validation in larger, independent cohorts.

In summary, despite the stated limitations, the present study provides clinically relevant descriptive data on the early experience of a newly established heart transplantation center and characterizes the clinical course of the first 100 consecutive transplant recipients from the perioperative period through 12 months of follow-up. The analysis provides information on early mortality, major post-transplant complications, rejection and infectious burden, and healthcare utilization in a real-world transplant population. The exploratory survival analyses should not be interpreted as definitive prognostic models; rather, they may help generate hypotheses regarding potentially relevant recipient- and donor-related factors that warrant evaluation in larger multicenter cohorts.

## 6. Conclusions

The first 100 heart transplantations performed at our newly established center were associated with 77% survival at 12 months. The first year after HTx was characterized by substantial clinical burden, including frequent rejection and infectious complications as well as intensive planned follow-up. Most deaths occurred early after transplantation. Exploratory analyses identified potential associations between 12-month mortality and pre-transplant CVP, troponin concentrations, and donor–recipient sex mismatch. These findings should be considered hypothesis-generating and require validation in larger, multicenter cohorts.

## Figures and Tables

**Table 1:** Baseline characteristics of the study population (*n* = 100).

Variable	Total
Age, years	54 (45–64)
Male sex	82 (82%)
BMI, kg/m^2^	26.1 (4.0)
Ischemic HF etiology	44%
Hypertension	46%
Hyperlipidemia	41%
Diabetes mellitus	26%
Chronic kidney disease/hemodialysis	30%
Bronchial asthma/COPD	4%
Smoking history/current smoking	43%
Hypothyroidism	18%
Hyperthyroidism	9%
Previous CABG	13%
Previous PCI	42%
Stroke/TIA	12%
AF/AFI/paroxysmal	47%
ICD/CRT-D	58%
Inotropic/vasopressor treatment	39%
Temporary MCS	20%
Durable LVAD (HeartMate 3)	7%
Elective transplant	18%
CVP, mmHg	9.0 (6.0–14.0)
Mean pulmonary artery pressure, mmHg	30.9 (9.9)
PCWP, mmHg	20.5 (15.5–25.0)
Cardiac output, L/min	4.1 (3.4–4.8)
Cardiac index, L/min/m^2^	2.1 (1.8–2.4)
PVR, WU	2.3 (1.8–3.3)
WBC, ×10^3^/µL	8.36 (6.42–11.49)
Hemoglobin, g/dL	12.85 (10.55–14.25)
Platelets, ×10^3^/µL	193 (149–238)
Creatinine, mg/dL	1.09 (0.94–1.39)
eGFR-MDRD, mL/min/1.73 m^2^	66.5 (53.0–84.0)
Albumin, g/dL	3.50 (2.90–4.00)
CRP, mg/L	10.1 (3.2–51.3)
Troponin, ng/L	23.6 (11.4–214.5)
NT-proBNP	4035 (1852–7962)

Continuous variables are presented as mean (SD) or median (IQR), categorical variables are presented as n (%). Abbreviations: AF/AFI, atrial fibrillation/atrial flutter; BMI, body mass index; CABG, coronary artery bypass grafting; COPD, chronic obstructive pulmonary disease; CRP, C-reactive protein; CRT-D, cardiac resynchronization therapy-defibrillator; CVP, central venous pressure; eGFR, estimated glomerular filtration rate; HF, heart failure; ICD, implantable cardioverter-defibrillator; LVAD, left ventricular assist device; MCS, mechanical circulatory support; NT-proBNP, N-terminal pro-B-type natriuretic peptide; PCI, percutaneous coronary intervention; PCWP, pulmonary capillary wedge pressure; PVR, pulmonary vascular resistance; TIA, transient ischemic attack; WBC, white blood cell count.

**Table 2:** Perioperative and in-hospital outcomes after heart transplantation.

Outcome	Total (*n* = 100)
Nitric oxide	54%
IABP	14%
ECMO	14%
Impella	0%
Right ventricular failure	2%
Renal replacement therapy	44%
In-hospital mortality	17%
Median time to death, days	17 (3–78)
Hospital stay among survivors, days	43
ICU stay among survivors, days	7

Abbreviations: ECMO, extracorporeal membrane oxygenation; IABP, intra-aortic balloon pump; ICU, intensive care unit.

**Table 3:** Selected laboratory parameters during follow-up after heart transplantation.

Parameter	1 Week (*n* = 100)	1 Month (*n* = 100)	3 Months (*n* = 83)	6 Months (*n* = 79)	12 Months (*n* = 77)
WBC, ×10^3^/µL	17.5 (14.1–21.0)	10.7 (7.9–13.1)	6.89 (5.71–8.98)	6.83 (4.94–8.18)	6.86 (6.00–8.45)
Hemoglobin, g/dL	10.0 (9.5–10.5)	10.9 (10.0–11.9)	11.6 (10.8–12.5)	12.3 (11.1–13.7)	12.9 (11.7–14.2)
Platelets, ×10^3^/µL	134 (101–180)	235 (164–323)	253 (213–298)	220 (173–276)	210 (178–250)
Creatinine, mg/dL	1.44 (1.06–1.80)	1.08 (0.82–1.60)	1.15 (0.89–1.47)	1.16 (0.95–1.42)	1.23 (1.03–1.54)
eGFR-MDRD, mL/min/1.73 m^2^	54.0 (40.0–77.5)	71.5 (45.0–101.0)	68.8 (50.0–88.5)	65.5 (54.5–84.5)	66.0 (45.5–78.3)
Albumin, g/dL	3.20 (2.95–3.40)	3.20 (2.90–3.50)	4.10 (3.40–4.30)	4.20 (3.90–4.50)	4.45 (4.30–4.70)
CRP, mg/L	48.5 (29.5–70.9)	30.1 (14.2–50.7)	9.35 (3.48–23.78)	3.66 (1.52–19.51)	2.19 (1.22–4.86)
Troponin, ng/L	8175 (4621–15,411)	228 (90–716)	17.5 (10.7–35.1)	9.10 (4.20–16.20)	5.98 (2.35–13.98)
NT-proBNP	—	—	1623 (854–4413)	849 (358–1840)	447 (208–774)

Values are presented as median (IQR). Abbreviations: CRP, C-reactive protein; eGFR, estimated glomerular filtration rate; NT-proBNP, N-terminal pro-B-type natriuretic peptide; WBC, white blood cell count.

**Table 4:** Clinical outcomes during 3-, 6-, and 12-month follow-up.

Outcome	3 Months (*n* = 85)	6 Months (*n* = 81)	12 Months (*n* = 77)
Infectious complications	51%	15%	14%
Heart failure decompensation	3%	3%	1%
RRT	6%	1%	0%
Patients with at least one episode of clinically significant acute cellular rejection (grade ≥ 3) at each follow-up time point	16%	8%	7%
All-cause mortality	15%	19%	23%
NYHA class < II	97%	97%	93%

Abbreviations: NYHA, New York Heart Association; RRT, renal replacement therapy.

**Table 5:** Pharmacotherapy and immunosuppressive therapy during follow-up.

Medication	Baseline (*n* = 100)	6 Months (*n* = 79)	12 Months (*n* = 77)
Tacrolimus	—	95%	94%
Mycophenolate mofetil	—	88%	93%
Cyclosporine	—	5%	6%
Everolimus	—	1%	1%
Corticosteroids	—	99%	33%
ACE inhibitor	41%	46%	54%
ARB	4%	3%	4%
Beta-blocker	82%	31%	39%
MRA	70%	3%	3%
Statin	58%	95%	97%
SGLT2 inhibitor	49%	19%	19%
Loop diuretic	75%	30%	17%
Thiazide/thiazide-like diuretic	11%	3%	3%
PPI	60%	97%	89%
Acetylsalicylic acid	18%	85%	81%
Anticoagulation	68%	7%	13%

Abbreviations: ACE, angiotensin-converting enzyme; ARB, angiotensin II receptor blocker; MRA, mineralocorticoid receptor antagonist; PPI, proton pump inhibitor; SGLT2, sodium-glucose cotransporter-2.

**Table 6:** Hospitalizations and selected clinical parameters during follow-up.

Parameter	3 Months (*n* = 83)	6 Months (*n* = 79)	12 Months (*n* = 77)
Outpatient visits per patient	0.28 ± 0.62	0.35 ± 0.71	0.49 ± 0.91
Planned hospitalizations per patient	3.20 ± 1.07	1.60 ± 0.59	2.93 ± 0.52
Unplanned hospitalizations per patient	0.19 ± 0.48	0.095 ± 0.294	0.14 ± 0.49
Weight, kg	74.5 ± 13.0	77.0 ± 13.0	81.1 ± 13.7
BMI, kg/m^2^	22.8 ± 6.9	25.0 ± 3.7	26.7 ± 4.0
Heart rate, beats/min	92.6 ± 10.7	93 ± 10.2	91 ± 8
Systolic arterial pressure, mmHg	129.0 ± 16.4	131 ± 15.8	135 ± 16
Diastolic arterial pressure, mmHg	82.1 ± 8.9	82 ± 9.8	86 ± 9

Values are presented as mean ± SD. Abbreviations: BMI, body mass index; SD, standard deviation.

**Table 7:** Cox proportional hazards regression models for all-cause mortality within 12 months after heart transplantation.

Model	Covariate	*p*-Value	Hazard Ratio (HR)	95% CI
1	Ischemic etiology	0.117	1.997	0.840–4.744
1	Troponin	0.038	2.543	1.053–6.141
1	Albumin	0.127	0.480	0.187–1.234
1	CVP	0.005	5.180	1.638–16.379
2	Troponin	0.054	2.330	0.984–5.519
2	Albumin	0.066	0.421	0.167–1.060
2	CVP	0.004	5.202	1.708–15.846
2	Donor–recipient sex mismatch	0.081	2.273	0.903–5.719
3	Troponin	0.075	2.176	0.923–5.129
3	Albumin	0.106	0.475	0.193–1.170
3	Donor–recipient sex mismatch	0.026	2.834	1.130–7.105
3	Ischemic etiology	0.036	2.565	1.064–6.183
4	Albumin	0.055	0.411	0.166–1.018
4	Donor–recipient sex mismatch	0.029	2.736	1.106–6.769
4	Ischemic etiology	0.072	2.247	0.931–5.421
4	CVP	0.010	4.151	1.416–12.173
5	Donor–recipient sex mismatch	0.119	2.115	0.825–5.420
5	Ischemic etiology	0.087	2.129	0.896–5.059
5	CVP	0.013	3.854	1.335–11.126
5	Troponin	0.056	2.370	0.979–5.737

Abbreviations: CI, confidence interval; CVP, central venous pressure; HR, hazard ratio.

## Data Availability

The data presented in this study are available upon request from the corresponding author.

## Abbreviations

AdHF—advanced heart failure; AUC—area under the curve; BMI—body mass index; CI—confidence interval; CPET—cardiopulmonary exercise testing; CKD—chronic kidney disease; CRT—cardiac resynchronization therapy; CRP—C-reactive protein; CVP—central venous pressure; DAOH—days alive and out of hospital; ECMO—extracorporeal membrane oxygenation; HF—heart failure; HR—hazard ratio; HTx—heart transplantation; hs-cTnI—high-sensitivity cardiac troponin I; hs-cTnT—high-sensitivity cardiac troponin T; ICD—implantable cardioverter-defibrillator; IQR—interquartile range; ISHLT—International Society for Heart and Lung Transplantation; IHTSA—International Heart Transplant Survival Algorithm; LVAD—left ventricular assist device; MCS—mechanical circulatory support; MRA—mineralocorticoid receptor antagonist; NT-proBNP—N-terminal pro-B-type natriuretic peptide; PAP—pulmonary artery pressure; PCI—percutaneous coronary intervention; PVR—pulmonary vascular resistance; RHC—right heart catheterization; ROC—receiver operating characteristic; RRT—renal replacement therapy; SD—standard deviation; UNOS—United Network for Organ Sharing; WBC—white blood cell count.
